# Photosystem I: A Paradigm for Understanding Biological Environmental Adaptation Mechanisms in Cyanobacteria and Algae

**DOI:** 10.3390/ijms25168767

**Published:** 2024-08-12

**Authors:** Li-Rong Tian, Jing-Hua Chen

**Affiliations:** 1 Ministry of Education Key Laboratory of Molecular and Cellular Biology, Hebei Research Center of the Basic Discipline of Cell Biology, Hebei Collaboration Innovation Center for Cell Signaling and Environmental Adaptation, College of Life Sciences, Hebei Normal University, Shijiazhuang 050024, China; tianlr@hebtu.edu.cn; 2 College of Life Sciences, Zhejiang University, Hangzhou 310058, China

**Keywords:** photosystem I, LHCI, cyanobacteria, algae, environmental adaptation

## Abstract

The process of oxygenic photosynthesis is primarily driven by two multiprotein complexes known as photosystem II (PSII) and photosystem I (PSI). PSII facilitates the light-induced reactions of water-splitting and plastoquinone reduction, while PSI functions as the light-driven plastocyanin-ferredoxin oxidoreductase. In contrast to the highly conserved structure of PSII among all oxygen-evolving photosynthetic organisms, the structures of PSI exhibit remarkable variations, especially for photosynthetic organisms that grow in special environments. In this review, we make a concise overview of the recent investigations of PSI from photosynthetic microorganisms including prokaryotic cyanobacteria and eukaryotic algae from the perspective of structural biology. All known PSI complexes contain a highly conserved heterodimeric core; however, their pigment compositions and peripheral light-harvesting proteins are substantially flexible. This structural plasticity of PSI reveals the dynamic adaptation to environmental changes for photosynthetic organisms.

## 1. Introduction

Photosystem I (PSI) is a large pigment–protein complex that catalyzes the reactions including the light-induced electron transfer and the reduction of ferredoxin (Fd) at the early stage of oxygenic photosynthesis [1]. The basic functional unit of PSI consists of a core complex and its peripheral light-harvesting antenna. The core complex harbors three pairs of chlorophylls (Chls), two phylloquinones and three [4Fe-4S] clusters, which constitute the central electron transfer chain (ETC) [2]. Once the solar energy is absorbed by antenna pigments, it is transferred to the core complex and the excited energy is trapped at P_700_, a special pair of Chls, where charge separation happens. The generated free electrons are quickly transferred to one acceptor Chl (A_0_) and subsequently to the terminal electron acceptor Fd through cofactors along the ETC. Simultaneously, the oxidized P_700_ (P_700_^+^) is replenished with an electron from a soluble electron donor of either a plastocyanin or a cytochrome. The reduced Fd provides the reducing power for the generation of ATP and NADPH, both of which are used in the subsequent CO_2_ fixation reactions [3].

Due to its crucial role, the structure and function of PSI have been extensively studied [4]. For a long time, single-crystal X-ray diffraction (SC-XRD) was the most important technique for elucidating the structure of PSI, particularly in resolving the structures of cyanobacterial PSI core trimers and higher plant PSI-LHCI supercomplex [5,6,7,8,9]. The first atomic structure of cyanobacterial PSI was revealed by SC-XRD in 2001, which was composed of 12 protein subunits and 127 cofactors [5]. However, the short-comings of SC-XRD—the prerequisite of substantial amounts of highly purified protein samples and the necessity for their crystallization—limit its application in PSI study. This is particularly evident in the case of algal PSI which contains a large number of light-harvesting antenna subunits, and is difficult to form regular single crystals. In contrast, single-particle cryogenic electron microscopy (cryo-EM) does not suffer from these limitations. In the past decade, cryo-EM has developed rapidly, reaching the level of SC-XRD in structural analysis [10]. Many previously unanswered issues related to PSI have been addressed with a large number of PSI structures being determined by cryo-EM [11,12,13,14,15]. These structures provide a deeper understanding of the complexity and function of PSI, which is important for the self-regulation mechanisms of oxygenic photosynthetic organisms to cope with various environmental challenges such as light intensity fluctuations, far red light, iron-deficiency stress and salt stress [16,17].

This review focuses on the structural variations of PSI in photosynthetic microorganisms, including prokaryotic cyanobacteria and various eukaryotic algae, and discusses the molecular mechanisms of high-efficiency energy and electron transfer within different PSI complexes. Through an in-depth exploration of these variations, we aim to inspire new thinking about the key role of PSI and the adaptive mechanisms by which different photosynthetic organisms thrive in different ecological niches.

## 2. Structural Variations of PSI Complexes in Cyanobacteria

### 2.1. Oligomers of PSI Complexes in Cyanobacteria

**Figure 1:**
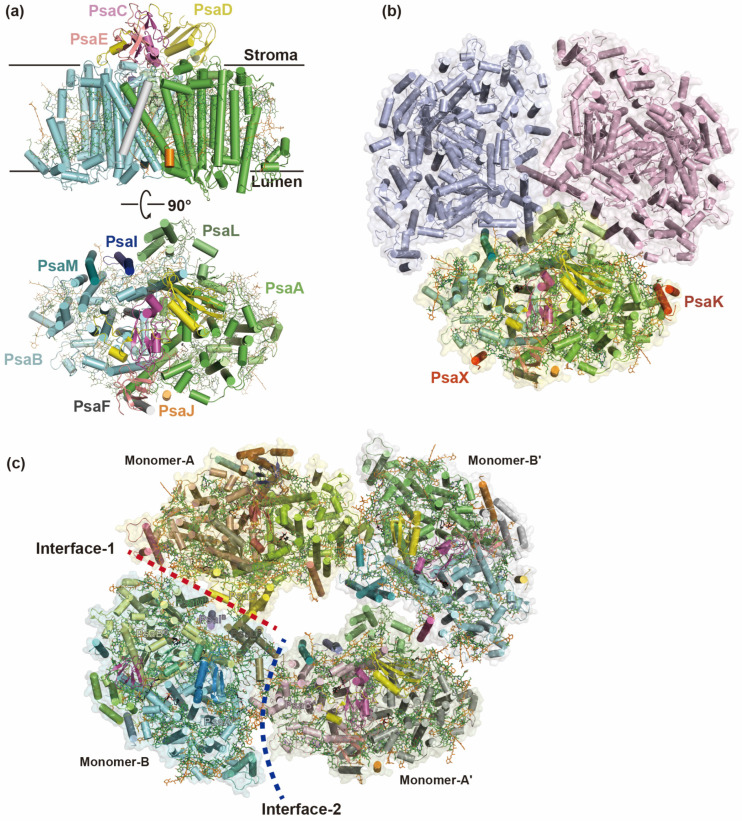
Structural diversity of cyanobacterial PSI. (**a**,**b**) The monomeric (PDB ID: 6LU1) and trimeric (PDB ID: 1JB0) PSI complexes from *T. elongatus* share similar subunit components; however, the PSI monomer lacks two peripheral subunits, PsaK and PsaX, which are colored in red. (**c**) The tetrameric PSI is mainly observed in heterocyst-forming cyanobacteria. A PSI tetramer (PDB ID: 6JEO) in *Anabaena* sp. PCC 7120 is organized with two PSI dimers with a *C*2 symmetry, forming two different interfaces (marked in dashed red and blue lines) between the neighboring PSI monomers.

In most cyanobacteria, the PSI monomers assemble into a stable trimeric structure with a 3-fold rotational symmetry [5,18] (Figure 1). Each PSI monomer consists of a highly conserved heterodimeric core (PsaA/B) and variable peripheral subunits (PsaC/D/E/F/I/J/K/L/M/X), depending on the species [18,19]. The majority of the cofactors are coordinated by the dimeric PsaA/B core, and the molecular mass of a PSI trimer exceeds 1 MDa [5]. Recently, owing to advancements in cryo-EM, structures of PSI monomers, tetramers and some heterogeneous complexes from cyanobacteria have been determined at near-atomic resolutions [20,21,22,23] (Figure 1).

The tetrameric PSI is widespread among heterocyst-forming cyanobacteria such as the mesophilic filamentous heterocyst-forming cyanobacterium *Anabaena* sp. PCC 7120 and the thermophilic non-heterocyst-forming cyanobacterium *Chroococcidiopsis* sp. TS-821, and their close relatives [23,24,25]. Physiological studies have shown that factors such as nitrogen source, temperature stress or salinity have no effect on the formation of PSI trimer or tetramers; however, under high light conditions, tetrameric PSI formation is favored and is accompanied by an increased content of novel carotenoids (Cars), like myxoxanthophyll, canthaxanthin, and echinenone [25]. Compared with the trimeric PSI, the four monomers within the PSI tetramer are structurally organized as a dimer of dimers with a *C*2 symmetry, forming two distinct interfaces (Figure 1). The two attached PSI dimers are stabilized to a large extent by electrostatic interactions of amino acid residues at the interfaces, as well as by the nearby Chls and specific lipids [26] (Figure 1).

The dimeric cyanobacterial PSI complexes can be identified by biochemical methods in *Thermosynechococcus elongatus* (*T. elongatus*) and *Anabaena* sp. PCC 7120 [18,23]; however, only the structure of the *Anabaena* PSI dimer has been reported, which is the same as one of the dimers in a PSI tetramer [20] (Figure 1). Low-resolution structures of monomeric PSI complexes have only recently been reported [16,27] (Figure 1). The PSI monomer shows no significant difference in NADP^+^ reduction rates compared to the PSI trimer [27]; however, the fluorescence spectrum of monomeric PSI exhibits a significant blue shift, which is caused by the loss of several peripheral red Chls [16]. Another notable difference lies in the disruption of the short C-terminal α-helix of PsaL after monomerization [18,27]. This short α-helix is believed to function as a critical factor in the formation and stabilization of trimeric and tetrameric PSI complexes [19,28,29,30].

### 2.2. PSI-IsiA Complexes in Iron-Deficient Environment

**Figure 2:**
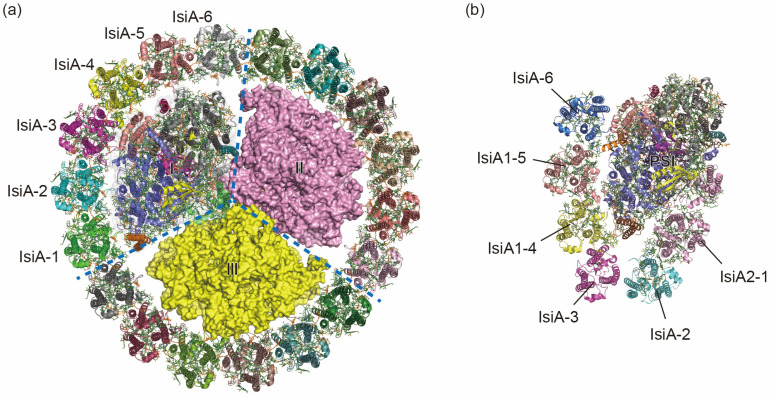
Two structures of cyanobacterial PSI–IsiA complexes under iron-deficiency condition. (**a**) The mesophilic cyanobacterium *Synechocystis* sp. PCC 6803 forms PSI3–IsiA18 supercomplex (PDB ID: 6K33) with three-fold rotational symmetry. (**b**) The monomer–PSI–IsiA6 complex of *Anabaena* sp. PCC 7120 (PDB ID: 7Y3F) associates six IsiA subunits. The C-terminal PsaL-like domain of IsiA2-1 occupies the corresponding position of PsaL in the *Anabaena* PSI tetramer.

Over the long period of evolution, cyanobacteria have developed special antenna systems that enable them to thrive under diverse environmental pressures, notably low iron, intense light, and oxidative stress [31,32,33,34,35]. These antenna systems primarily comprise two types: the membrane-bound iron-stress-induced A proteins (IsiAs) and the prochlorophyte Chl *a*/*b*-binding (Pcb) protein family, and the water-soluble phycobilisomes [36,37]. Both of the IsiA/Pcb families possess six transmembrane helices and exhibit similar structural features with the CP47 and CP43 subunits of PSII [38,39,40] (Figure 2). Under normal growth conditions, in order to achieve efficient absorption and utilization of light, cyanobacteria form giant complexes of PSI–phycobilisome and PSII–phycobilisome, whose structures have recently been resolved [41,42,43].

In the cyanobacterium *Synechocystis* sp. PCC 6803, IsiA proteins can congregate in clusters of up to 18 copies surrounding a trimeric PSI core, forming a PSI3–IsiA18 supercomplex [44,45] (Figure 2). The binding of IsiA complexes enhances the absorption cross-section, thereby compensating for the diminished PSI levels within the membrane [46,47,48]. The IsiA protein was primarily proposed to serve as an excess energy quencher and/or a reservoir for Chls, facilitating its subsequent integration into the photosystems [49,50,51]. However, a high-resolution structure and time-resolved fluorescence spectra of the PSI–IsiA complex from a thermophilic cyanobacterium *Thermosynechococcus vulcanus* showed clear excitation-energy transfer from IsiA to PSI, strongly indicating that the IsiA protein mainly functions as an energy donor but not an energy quencher within the complex [35]. When cultured in iron-deprivation environments, the protein Flavodoxin (Fld) takes over the role of Fd, accepting electrons from one of the [4Fe-4S] clusters coordinated by the PSI subunit PsaC [52]. The structure of the PSI3-IsiA18-Fld3 complex from *Synechococcus* sp. PCC 7942 reveals that three Fld molecules bind symmetrically to the trimeric PSI core [31]. Within each PSI monomer, Fld binds to the surface of PSI by electrostatic action [31].

The number of *isiA* genes varies among different species of cyanobacteria. Cyanobacterium *Anabaena* sp. PCC 7120 has four types of *isiA* genes: *isiA1*, *isiA2*, *isiA3*, and *isiA5* [33]. However, the structures of the IsiA proteins identified in the PSI–IsiA complexes from *Synechocystis* sp. PCC 6803, *Synechococcus elongatus* PCC 7942, and *Thermosynechococcus vulcanus* NIES–2134 are similar to that of IsiA1 from *Anabaena* [31,32,35]. In *Anabaena*, the PSI monomer–IsiA complex consists of six IsiA subunits, five of which contain six transmembrane helices that bind Chls and Cars [34] (Figure 2). The remaining IsiA subunit (IsiA2) adjacent to PsaD/I has nine transmembrane helices and exhibits a remarkable structural correspondence with PsaL, particularly in the C-terminal domain, which may substitute for the role of PsaL in the *Anabaena* PSI tetramer [34] (Figure 2).

### 2.3. PSI Complexes from Chls d/f-Containing Cyanobacteria

Chlorophylls are crucial for energy capture, transfer, and charge separation in photosynthesis [53,54]. Photosynthetic organisms have evolved a variety of Chls with varied molecular structures and absorption ranges to achieve efficient light utilization [55]. Most oxygenic photosynthesis organisms possess Chls *a* within their photosynthetic apparatus [56]; however, other types of Chls, such as Chls *b*, *c*, *d*, and *f*, are found in specific lineages, which enables organisms to occupy unique ecological niches [54,57,58]. Unlike Chls *a*, *b* and *c*, which mainly absorb higher-energy light [59], Chls *d* and *f* absorb lower-energy light [60,61,62]. A mixture of different types of Chls broaden the absorption spectrum and promote the light utilization efficiency [56].

**Figure 3:**
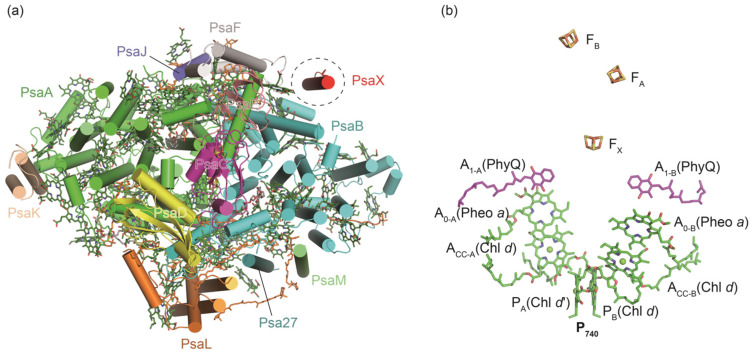
Structure and the cofactor arrangement along the electron transfer chain (ETC) of *A. marina* PSI monomer (PDB ID: 7DWQ). (**a**) The protein subunits are colored respectively, and the missing subunit PsaK is marked with dashed circle. (**b**) The cofactors of P_740_, A_cc_, A_0_, and A_1_ of the ETC are arranged in two separated branches (branch A and branch B). Phylloquinones and Fe4S4 clusters are labeled as PhyQ, F_X_, F_A_, and F_B_, respectively. Uniquely, the Chls of A_0_ in *A. marina* PSI are identified as two pheophytins *a*, instead of the typical Chls *a* in other cyanobacterial PSI complexes [53].

*Acaryochloris marina* (*A. marina*) is a unique cyanobacterial species which uses *d*-type Chls as its dominant photosynthetic pigments and is capable of using far-red light to drive oxygenic photosynthesis [63,64,65]. The structure of *A. marina* PSI has been determined with resolutions of 2.58 Å and 3.3 Å, respectively [66,67]. The overall structure of *A. marina* PSI resembles the PSI trimers from other cyanobacteria; however, the peripheral subunits of PsaI and PsaX are missing (Figure 3a). A novel subunit, Psa27, identified in *A. marina* PSI, exhibits a similar structure and location with PsaI of *T. elongatus* PSI, indicating their similar functions in stabilizing the PSI trimer [67] (Figure 3a). The total number of pigments (Chls and carotenes) in *A. marina* PSI is less than that in *T. elongatus* PSI, and most of the missing pigments are located in the peripheral small subunits PsaJ, PsaF, PsaM (Figure 3a). Uniquely, the paired Chls (known as P_740_) along the ETC of *A. marina* PSI is a dimer of Chl *d* and its epimer Chl *d*′, and the primary electron acceptor A_0_ is pheophytins *a*, rather than Chl *a*, which is found in other PSI structures [66,67] (Figure 3b).

The Chl *f*-containing cyanobacteria demonstrate a remarkable capacity for photosynthesis in the far-red and near-infrared regions [63,68,69]. However, Chl *f* is only induced under far-red light conditions and accounts for approximately 10% of the total Chls, indicating their specific roles within the photosystem [70,71]. The structures of PSI complexes from *Halomicronema hongdechloris* (*H. hongdechloris*) and *Fischerella thermalis* PCC 7521 grown under far-red light have been resolved [53,72]. The *H. hongdechloris* PSI binds 83 Chls *a* and 7 Chls *f*, with all Chls *f* located at the periphery of PSI and excluded from the electron transfer chain, which suggests that Chls *f* function to harvest far-red light and enhance the uphill energy transfer [53]. Further studies demonstrate that far-red light induces extensive remodeling of the photosynthetic apparatus in *H. hongdechloris* by altering the expression of genes encoding PSI core subunits and by modifying the types of pigments associated with PSI [73,74,75]. Indeed, differences in the sequences of several core subunits, including PsaA, PsaB, PsaI, and PsaL, have been observed in the PSI complex under far-red light compared to that under white light [66,67].

## 3. Structural Variations of Algal PSI–LHCI Complexes

### 3.1. PSI–LHCI Complexes of Chlamydomonas reinhardtii

**Figure 4:**
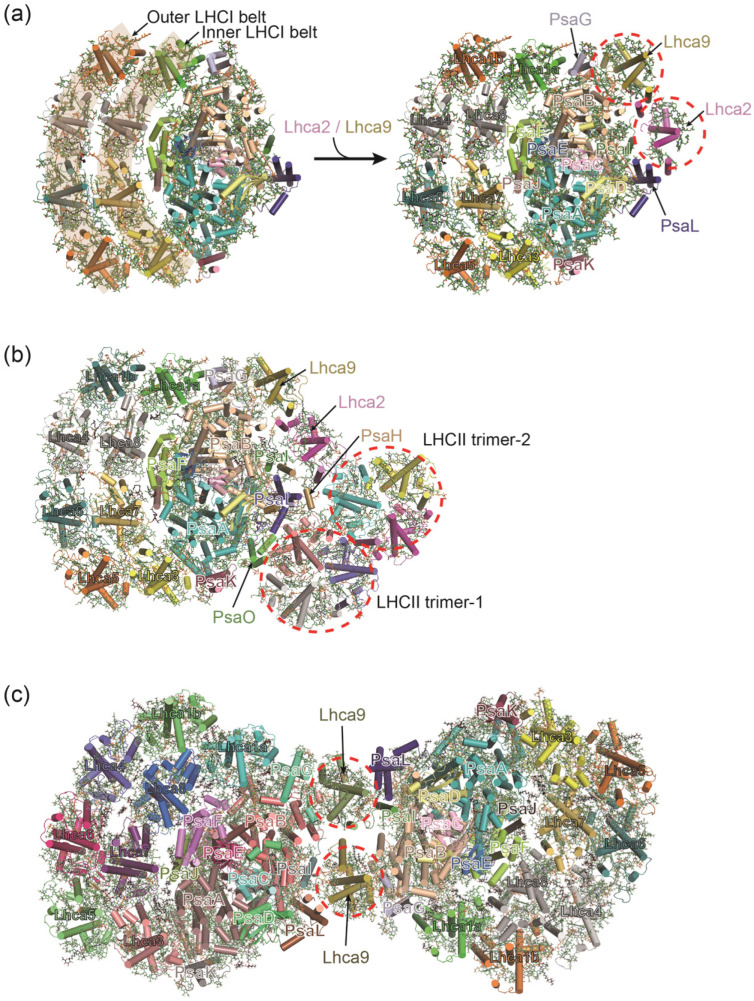
Structural variations of *C. reinhardtii* PSI–LHCI complexes. (**a**) The structures of *C. reinhardtii* PSI–LHCI complexes binding with eight and ten Lhca subunits. The antenna subunits are distributed as three belts: two crescent-shaped belts including one inner belt and one outer belt which are associated to one side of the PSI core (PDB ID: 6JO6), and an additional small belt made up of the Lhca2 and Lhca9 heterodimer on the opposite side (PDB ID: 6JO5). (**b**) During the state transitions (state 2), phosphorylated LHCIIs dissociate from PSII and migrate to PSI, forming the PSI–LHCI–LHCII supercomplex (PDB ID: 7D0J). (**c**) Under low light and anoxic conditions, PSI–LHCI dimer is formed from two PSI–LHCI monomers that are arranged in a “head-to-head” manner. The PsaH and Lhca2 subunits are absent; instead, two Lhca9 subunits interact with the subunits of PsaI, PsaL, PsaG from two PSI–LHCI monomers (PDB ID: 7ZQD).

The unicellular eukaryotic green alga *Chlamydomonas reinhardtii* (*C. reinhardtii*) is a model organism for studying photosynthesis, and the structure and function of its photosystem have been extensively studied [76]. The overall structure of the *C. reinhardtii* PSI–LHCI complex is similar to that of plant PSI–LHCI, both of which are composed of a core complex and a peripheral antenna system [8]; however, the *C. reinhardtii* PSI combines more peripheral antenna subunits. As depicted in Figure 4, the 10 Lhca proteins of *C. reinhardtii* PSI are distributed in three belts: one inner belt (Lhca1a/Lhca8/Lhca7/Lhca3), one outer belt (Lhca1b/Lhca4/Lhca6/Lhca5) and a Lhca2–Lhca9 heterodimer [77]. The Lhca2–Lhca9 heterodimer loosely attaches to the PSI core at the opposite side from the other LHCI belts, which may provide a docking site for the cytochrome *b*_6_*f* complex and enhance the photosynthetic cyclic electron flow [77] (Figure 4). Notably, all the *C. reinhardtii* Lhca subunits have a conserved transmembrane structure with other LHC family members, while showing differences at the N and C terminals [77]. The huge and complicated pigment network enables *C. reinhardtii* cells to adapt to the changing light environment.

To optimize the photosynthetic efficiency in fluctuating light conditions, photosynthetic organisms like plants and algae have developed a short-term light adaptation mechanism called state transitions [78]. This process ensures a balanced distribution of excitation energy between the two photosystems, PSI and PSII, through dynamic relocation of the light-harvesting antenna complex II (LHCII) [79,80]. The state transitions are regulated by the redox state of the plastoquinone (PQ) pool: in state 1, when the PQ pool is oxidized, LHCII remains primarily associated with PSII, forming the PSII–LHCII complex; conversely, in state 2, upon reduction of the PQ pool, protein kinases (such as STN7 in higher plants or STT7 in green algae) are activated via the cytochrome *b*_6_*f* complex [81,82]. These kinases then phosphorylate LHCIIs, promoting their partial dissociation from PSII and migration to PSI, which triggers the formation of the PSI–LHCI–LHCII supercomplex, enhancing PSI’s light-harvesting capacity [83,84,85,86]. The high-resolution structure of the PSI–LHCI–LHCII supercomplex from *C. reinhardtii* has been elucidated, and all four types of LHCII are found to associate with PSI under state 2 conditions [85] (Figure 4). Two LHCII trimers are associated with PSI–LHCI at the PsaO–PsaL–PsaH–Lhca2 side: one (LHCII–1) attaches to the PSI core by PsaO, PsaH, and PsaL, the other one (LHCII–2) attaches to the Lhca2 and LHCII–1 subunits [85] (Figure 4). As PSII is generally considered to be more susceptible to oxidative damage than PSI under high light conditions, the transfer of LHCII to PSI helps to reduce the excessive accumulation of excitation energy in PSII, thereby reducing the oxidative damage of PSII [84,87,88,89]. However, if the energy absorbed by LHCII is excessively funneled to the PSI core, the resulting overactivation of PSI can trigger the production of reactive oxygen species (ROS), ultimately diminishing the overall photosynthetic efficiency [87].

Recently, the PSI–LHCI dimer has been isolated from *C. reinhardtii* cells grown in low light and anoxic conditions [90]. This unique oligomerization of PSI is formed by two PSI–LHCI monomers arranged head-to-head, containing 40 protein subunits and more than 600 cofactors [90]. Unlike the dimeric formation observed in cyanobacterial PSI tetramers, which is mainly due to the movement of the stromal helices of PsaL [22], *C. reinhardtii* PSI–LHCI dimer is formed by the interactions of four subunits: Lhca9, PsaI, PsaL, PsaG, and their associated pigments [90]. The PsaH and Lhca2 subunits observed in the PSI–LHCI monomer are absent in the *C. reinhardtii* PSI–LHCI dimer; instead, a second Lhca9 subunit occupies the corresponding space for PsaH and Lhca2, and binds to the subunits PsaG, PsaL and PsaI of two PSI–LHCI monomers [90] (Figure 4). This unique oligomeric state of PSI–LHCI in *C. reinhardtii* reflects the diversity of its adaptation mechanisms to different environmental conditions [90].

### 3.2. PSI–LHCI Complexes of Red Algae

**Figure 5:**
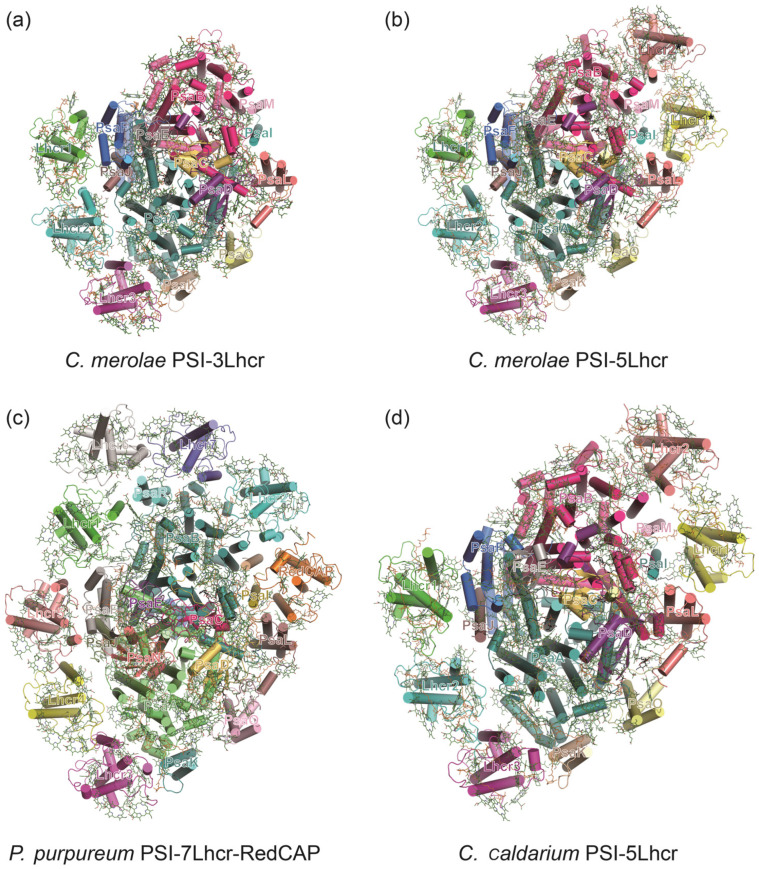
Comparison of the PSI–LHCI complexes from different red algae. (**a**,**b**) *C. merolae* contains two different forms of PSI–LHCI complexes, with one binding three Lhcr subunits (PDB ID: 5ZGH) and the other one binding five Lhcr subunits (PDB ID: 5ZGB). (**c**) *Porphyridium purpureum* PSI–LHCI associates seven LHCI subunits and one chlorophyll *a*/*b*-binding-like protein (RedCAP) (PDB ID: 7Y5E). (**d**) *Cyanidium caldarium* PSI–LHCI complex contains five LHCI subunits which are distributed as two separated clusters (PDB ID: 8WEY).

In addition to state transition, an alternative strategy that algae employ to regulate the antenna size of PSI is to change the number of associated LHCI subunits [91]. For red algae *Cyanidioschyzon merolae* (*C. merolae*), even when grown under optimal laboratory conditions, two PSI–LHCI complexes with antenna systems of different sizes can be isolated [92] (Figure 5). The smaller form (PSI–3Lhcr) binds three antenna subunits, which form an LHCR band and interact with PsaF, PsaJ, PsaA, and PsaK. The larger form (PSI–5Lhcr) binds two additional Lhcr proteins that are associated with the surface of PsaL, PsaI, PsaM, and PsaB, forming an additional LHCR* belt [92]. The major structural difference between PSI–5Lhcr and PSI–3Lhcr lies in the additional LHCR* belt; the counterpart core subunits and three Lhcr subunits of the canonical LHCR belt are identical [92]. Biochemical and spectroscopic data have revealed a close correlation between the ratio of these two forms and the light intensities in red algae [93]. Similarly, in the unicellular green algae *C. reinhardtii* and *Bryopsis corticulans* [91,94], the PSI core complex can associate with either 8 to 10 LHCI subunits, indicating that the binding state of LHCI and PSI in algae is flexible with environmental conditions [91]. Uniquely, the red alga *Porphyridium purpureum* PSI–LHCI contains seven LHCI subunits and one chlorophyll *a*/*b*-binding-like protein (RedCAP), and another red alga *Cyanidium caldarium* RK–1 belonging to the *Cyanidiophyceae* possesses seven or five LHCI subunits (Figure 5).

### 3.3. A Minimal PSI from Salt-Tolerant Green Alga Dunaliella salina

**Figure 6:**
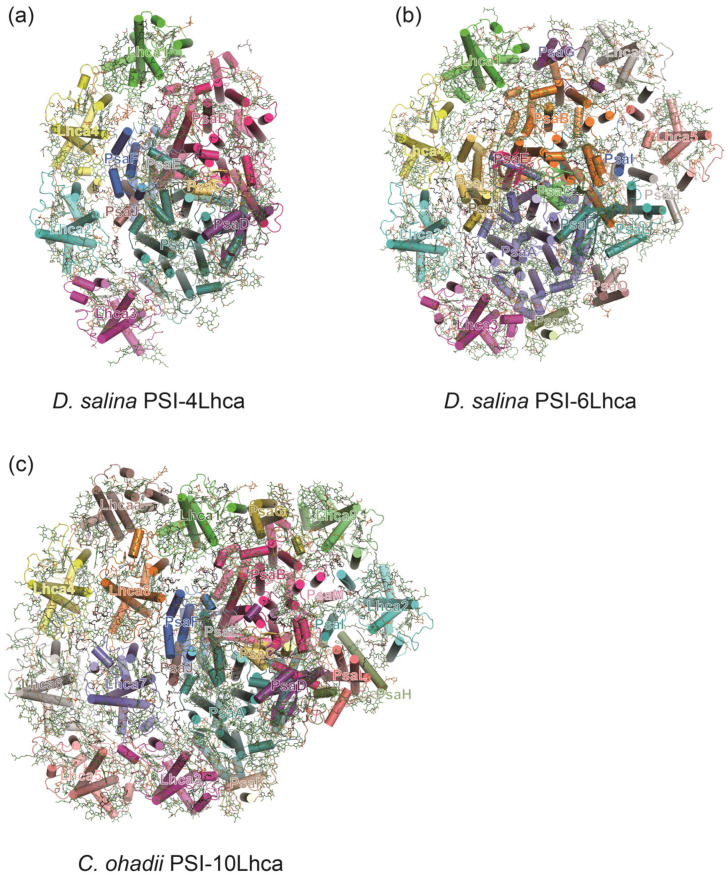
Structures of PSI–LHCI complexes from green algae *D. salina* (**a**,**b**) (PDB ID: 6QPH, 6SL5) and *C. ohadii* (PDB ID: 6ZZY) (**c**).

*Dunaliella salina* (*D. salina*) represents a unicellular green alga which can adapt to hypersaline environments and light stress. There exist two forms of PSI–LHCI in *D. salina*: a mini PSI–LHCI and a large PSI–LHCI [95]. The mini *D. salina* PSI–LHCI contains only seven PSI core subunits (PsaA–F and PsaJ) and four LHCI proteins (Lhca1–4) which are positioned at similar positions as the counterparts in plant PSI–LHCI [8,9] (Figure 6). The large *D. salina* PSI–LHCI contains 13 core subunits and 6 LHCI proteins (Lhca1–6), exhibiting a similar structure when compared to PSI–LHCI complexes derived from red algae and green algae [91,94]. However, the large *D. salina* PSI–LHCI lacks the second round of light-harvesting antenna (Figure 6). The different forms of *D. salina* PSI–LHCI complexes reveal different regulatory mechanisms of reducing the association of antenna proteins or forming distinct subunit interactions under certain physiological conditions.

### 3.4. PSI–LHCI in Desert Algae Chlorella ohadii

In the highly light-tolerant green algae *Chlorella ohadii* (*C. ohadii*), it has been observed that even when exposed to light intensities four times higher than those required for saturating CO_2_ fixation, the algae do not suffer from photodamage [96,97]. A comparative analysis of the PSI–LHCI structures from *C. ohadii* cells grown in low light (LL) and high light (HL) environments reveals that, to minimize photodamage, part of LHCI and the PSI core subunit, PsaO, are eliminated in PSI_HL_ [98]. The absence of the PsaO subunit indicates that the state transition is not triggered in *C. ohadii* under high light conditions (Figure 6). Another remarkable difference between PSI_LL_ and PSI_HL_ lies in the pigment composition and their number in LHCIs: approximately 50% of the Chls *b* in LHCI_HL_ are replaced by Chls *a* [98]. Notably, the highest substitution rates occur in the first LHCI_HL_ belt and the LHCI_HL_ dimer, both of which surround the PSI core complex [98]. This pigment substitution may lead to higher electron transfer rates within *C. ohadii* PSI_HL_, as PSI_HL_ exhibits a higher efficiency of photocurrent induction [98].

### 3.5. Diatom PSI-FCPI Complex

**Figure 7:**
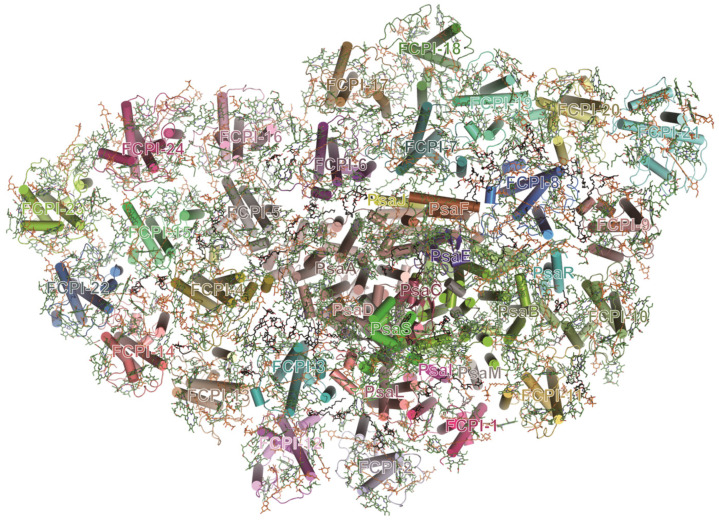
Subunit organization and pigment network of diatom *C. gracilis* PSI–FCPI complexes (PDB ID: 6LY5).

Diatoms are a large group of eukaryotic algae which account for about 20% of the global primary carbon fixation [99]. The PSI of diatoms is distinguished by a large number of light-harvesting subunits called fucoxanthin–Chl proteins (FCPIs), which bind Chl *a*/*c* instead of Chl *b* and fucoxanthin instead of lutein [100]. The PSI-FCPI complex from the diatom *Chaetoceros gracilis* shows an asymmetrical heart-shaped structure, consisting of 12 core subunits and 24 FCPIs, and associating with 326 Chls *a*, 34 Chls *c*, 102 fucoxanthins, 35 diadinoxanthins, 18 β-carotenes and other cofactors [101] (Figure 7). The structure of the diatom PSI core is conserved with that of cyanobacterial PSI; however, the subunit of PsaK is missing and two new subunits (PsaR and PsaS) are present [101]. The large FCPI antenna around the PSI core could be divided into three layers: an innermost layer, a semi-ring middle layer and an outermost layer [101]. Each FCPI subunit exhibits distinctive structures with different pigment compositions, and these FCPI subunits interweave with the PSI core to form a sophisticated pigment–protein network for the efficient light capture and energy transfer [101].

### 3.6. Tetrameric PSI from Glaucophyte Algae

**Figure 8:**
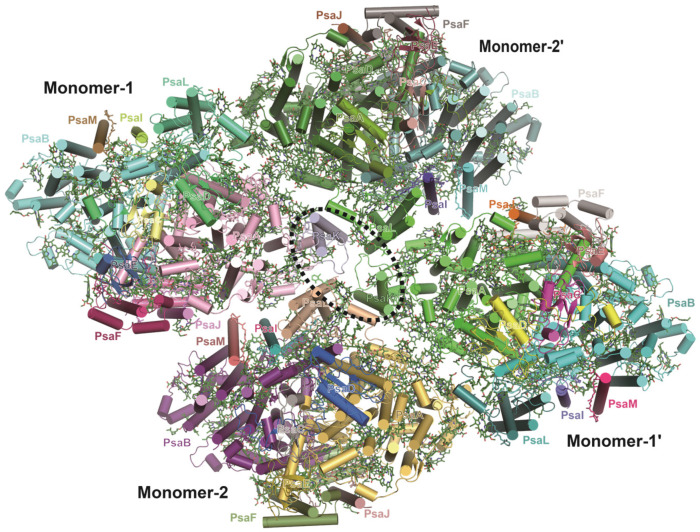
Structure of PSI tetramer from *Cyanophora paradoxa* (PDB ID: 7DR2).

Glaucophyte alga is a unique photosynthetic eukaryote that has plastid-like organelles termed cyanelles [102]. The cryo-EM structure of PSI from the glaucophyte alga *Cyanophora paradoxa* is determined as a tetramer; however, it is remarkably different from the previously observed tetrameric PSI from cyanobacteria in subunit composition and organization [103] (Figure 8). The *Cyanophora* PSI tetramers are composed of two types of structurally similar PSI monomers, termed as monomer 1 and monomer 2, the latter of which lacks the PsaK subunit [103] (Figure 8). A monomer 1 attaches to a monomer 2, creating a monomer 1–monomer 2 heterodimer, and two such heterodimers further give rise to a tetramer in an inverse parallel manner [103] (Figure 8). Due to the unique assembly, the monomer–monomer interactions as well as the excitation-energy transfer among Chls in *Cyanophora* PSI tetramers are entirely different from those in cyanobacteria PSI tetramers [103] (Figure 1). The distinctive structural features of *Cyanophora* PSI highlight a vital evolutionary transition in photosynthetic machineries, illustrating an intermediary phase in the transformation from oligomeric forms to monomeric units within this early eukaryotic alga [103].

### 3.7. PSI-ACPI Complex in Cryptophytes

**Figure 9:**
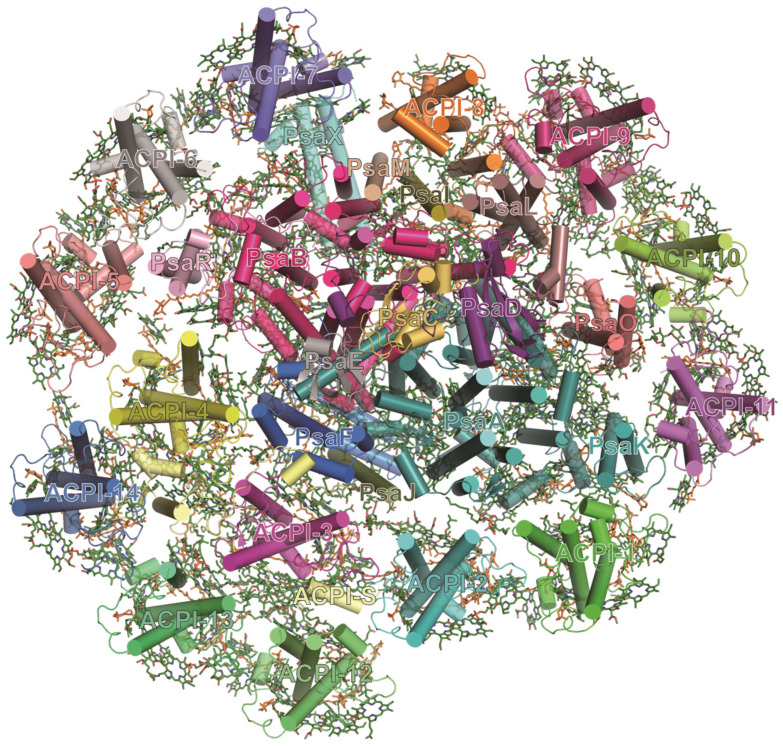
Structures of PSI–ACPI supercomplexes from cryptophyte *C. placoidea* (PDB ID: 7Y7B).

Cryptophytes (also called Cryptomonads or Cryptophyceae) are ancestral photosynthetic organisms that evolved through secondary endosymbiosis possibly between a red alga-like organism and a heterotrophic host [104,105,106]. Cryptophyte cells perform efficient oxygenic photosynthesis through their membrane-imbedded alloxanthin–Chl *a*/*c*-binding proteins (ACPs) and soluble phycobiliproteins as light-harvesting antennas [107]. The PSI–ACPI supercomplex from the cryptophyte *Chroomonas placoidea* consists of a monomeric PSI core and a huge peripheral antenna [108] (Figure 9). The PSI core contains 14 subunits, including 12 red algae-originated subunits, one diatom PsaR homolog, and one loosely associated extrinsic subunit (Unk1) [108]. The outer antenna surrounding the PSI core is made up of 14 ACPI subunits which are distributed in two layers: the inner layer has 11 ACPIs, and the outer layer has 3 ACPIs [108] (Figure 9). The association and energy transfer between the outer and inner ACPIs are mediated by a 20.6-kDa pigment-binding subunit termed ACPI-S [108] (Figure 9). However, the structure and pigment-binding sites of ACPI–S are different from the typical ACPI subunits [108]. Specifically, all ACPI apoproteins contain three major TM helices (αA, αB, αC) and an additional amphipathic helix (αD or αE). By contrast, ACPI–S has only one transmembrane helix and an amphipathic helix, but contains several long-terminal loops [108].

### 3.8. PSI-A_CP_PCI Complex in Symbiotic Dinoflagellates

**Figure 10:**
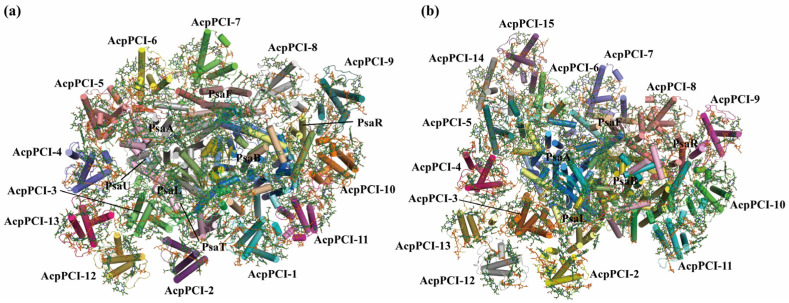
Structures and organizations of PSI-AcpPCI supercomplexes with 13 (**a**) and 14 (**b**) AcpPCIs from symbiotic dinoflagellate in *Symbiodinium*.

Dinoflagellates constitute a significant proportion of unicellular eukaryotes, inhabiting diverse aquatic habitats [109,110,111]. Many dinoflagellate species could form symbionts with invertebrates such as corals, or with algae-like diatoms [112,113,114]. The well-known symbiosis between corals and photosynthetic dinoflagellates of the *Symbiodiniaceae* family is instrumental in the development and sustenance of coral reefs [112]. The PSI–LHCI supercomplex from *Symbiodinium* comprises a core complex and a unique peripheral antenna system, showing similar structural features with red algal PSI–LHCR, cryptophyte PSI–ACPI, and diatom PSI–FCPI, but exhibiting specific characteristics in the protein organization [115,116,117] (Figure 10). In particular, the PSI core consists of 13 subunits including 2 new-found extrinsic subunits, PsaT and PsaU [117] (Figure 10). The overall structure of *Symbiodinium* PSI core is similar to that of the diatom PSI core, but the PsaK and PsaO subunits are missing [117] (Figure 10). Remarkable differences emerge as modifications to the extrinsic loop regions of the PsaA and PsaB subunits and alterations in the C-terminal regions of several subunits including PsaD/E/I/J/L/M/R [116,117] (Figure 10). The peripheral antenna system of *Symbiodinium* PSI is composed of 13~14 peridinin–Chl *a*/*c*-binding light-harvesting antenna proteins (AcpPCIs), which are distributed in two layers around the PSI core [116,117] (Figure 10). Most of the pigment-binding sites in *Symbiodinium* PSI–AcpPCI are conserved with those in diatom PSI–FCPI, but there are some notable differences. Although *Symbiodinium* PSI–AcpPCI contains a small number of pigments and antenna subunits, the extended end domains of the PSI core and antenna subunits enable efficient protein interactions and intermolecular energy transfer [116,117]. Interestingly, in a red tidal dinoflagellate *Amphidinium carterae*, both the structures of PsaA/B subunits exhibit substantial shortenings and have more short loops, leading to a reduction of over 20 pigment-binding sites compared with those of diatom PsaA/B subunits [116]. However, the other core subunits, including PsaD/F/I/J/L/M/R, show significant elongations and additional pigment-binding sites compared with those in diatom PSI [116]. Additionally, the *Amphidinium carterae* PSI core is associated with 18 AcpPCIs that bind a large number of xanthophyll cycle Cars, which may compensate for the smaller PsaA/B subunits [116].

## 4. Perspective

The precise structural determination of photosynthetic protein complexes is crucial for revealing the mechanisms of their efficient working modes. As more and more PSI structures from different species are resolved, we are able to gain a more complete understanding of how this complex protein machine works. Here, we discussed the structural and functional characteristics of various PSI complexes and their associated light-harvesting proteins in cyanobacteria and algae, reflecting their remarkable plasticity of environmental adaptation under different conditions.

However, it should be noted that photosynthetic organisms respond to environmental stress at different levels and in different ways. These stresses, including extreme temperatures, limited nutrient or water supply, and salinity, could trigger photoinhibition which in turn cause a loss in energy conversion efficiency and photosynthetic capacity. For example, under high light and low CO_2_ conditions, the paired chlorophylls (P_700_) in the PSI core adopt an oxidized state. This state serves to modulate light utilization and dissipate excess excited energy within PSI. Keeping P_700_ in an oxidized state is a crucial strategy for protecting PSI against potential photodamage [118,119]. The mechanisms for preventing photoinhibition in PSI have been extensively investigated from plants and photosynthetic organisms. Notably, the PGR5-dependent cyclic electron transfer (CET) plays a preferential role in the acceptor-side regulation of PSI, which is necessary for PSI photoprotection by facilitating the oxidation of P_700_ under high light [119,120]. For an in-depth review of PSI photoprotection, please refer to references [121,122,123].

Given its pivotal role in oxygenic photosynthesis, the structural diversity of PSI is a consequence of billions of years of biological evolution. For eukaryotic photosynthetic organisms (such as algae and plants), they tend to evolve more complex light-harvesting systems and finer energy regulation mechanisms (such as the state transition mechanism), while in prokaryotic photosynthetic organisms (such as cyanobacteria), there are different aggregation forms of reaction centers. Across all oxygen-evolving photosynthetic organisms, PSI is characterized by a structurally conserved heterodimeric core. This core serves as a critical binding platform for the majority of PSI’s cofactors, establishing the foundational architecture essential for the complex processes of charge separation and electron transport within the photosynthetic machinery.

In recent years, the structures of photosystems from non-oxygen-producing photosynthetic bacteria, including heliobacteria, green sulfur bacteria and Acidobacteria, have been resolved [124,125,126]. The photosynthetic reaction centers of these bacteria are in the forms of homodimers, which are structurally highly conserved with the PSI core of oxygen-producing photosynthetic organisms, but lack complex light-trapping protein subunits within the membrane. Instead, efficient absorption of light energy is mainly achieved through specific extracellular light-trapping systems, such as the chlorosome.

## Data Availability

Not applicable.
